# Knowledge, attitudes and practices of community pharmacists on generic medicines in Palestine: a cross-sectional study

**DOI:** 10.1186/s12913-017-2813-z

**Published:** 2017-12-28

**Authors:** Naser Y. Shraim, Tasneem A. Al Taha, Rawan F. Qawasmeh, Hiba N. Jarrar, Maram A. N. Shtaya, Lama A. Shayeb, Waleed M. Sweileh

**Affiliations:** 1 Department of Pharmacy, College of Medicine and Health Sciences, An-Najah National University, Nablus, Palestine; 2 Department of Pharmacology and Toxicology, College of Medicine and Health Sciences, An-Najah National University, Nablus, Palestine

**Keywords:** Generic medicines, Community pharmacists, Knowledge, Attitude, Practice, Palestine

## Abstract

**Background:**

Generic substitution in several countries has become a common practice. Besides, it is considered as a major cost minimizing strategy meant to contain pharmaceutical expenditure without compromising healthcare quality. However, the safety and quality issues of generic products are of top concerns of general practitioners and health work professionals. This study aimed to investigate community pharmacist’s knowledge, attitudes and practices toward generic medicines in Palestine.

**Methods:**

This study was a cross-sectional observational study employing a self-administered questionnaire. The questionnaire was of four main sections: demographic and practice details of the participants, knowledge, attitudes and the influencing factors related to selection and dispensing of generic medicines. A convenience sampling technique was implemented in this study in which the data collection form was distributed in West Bank- Palestine among a set of practicing pharmacists. Mann-Whitney-U or Kruskal-Wallis tests were used to comparison of different issues as appropriate. *P*-values of <0.05 were considered significant.

**Results:**

A total of 302 community pharmacists were interviewed, slightly more than half were males (52.3%). The mean knowledge score of participants regarding generic medicines was (5.91 ± 1.27) where the highest score was 8 of 10. Knowledge score was not significantly influenced by any of the socio-demographic characteristics. Our data showed that most of included pharmacists in the study (95.4%) agreed that health authorities should implement bioequivalence policies prior to marketing approval of generics, while 87.4% of participants agreed that they should be given the right to substitute generics and the majority (62.3%) support generic substitution for brand name drugs in all cases when a generic is available The main two factors affect pharmacists’ selection and dispensing of generic medicines are personal faith in the product (86.1%) and cost effectiveness of generic medicines (84.1%).

**Conclusions:**

Generic medicines substitution among pharmacists is widespread and prevalent. Our data found that participant pharmacists in Palestine had basic knowledge with regards to generic medicine. However, their knowledge score pertaining the technical and regulatory aspects of bioequivalence and pharmacokinetic parameters in particular was insufficient.

**Electronic supplementary material:**

The online version of this article (10.1186/s12913-017-2813-z) contains supplementary material, which is available to authorized users.

## Background

Generic versions of drug are very interesting component of the pharmaceutical market. They can only be produced after a patent expire of the branded drug [1–3]. According to the United States Food and Drug Administration (FDA), generic drug is defined as a drug that has the same characteristics as a branded drug in active ingredient, strength, route of administration, safety, dosage form, performance, quality and intended use. Generic drugs need to be bioequivalent to the branded drug to be licensed for marketing [4]. Although, biosimilar products are generic versions of biologics, significant differences exist between biosimilars and typical generic drugs. Chemical methods are generally used to produce generic drugs whereas biologicals are synthesized usually by cells or living organisms. This difference in origin leads to differences in structure, composition, manufacturing methods and equipment, intellectual property, formulation, handling, dosing, regulation, and marketing aspects [5].

Generic substitution is considered as a major cost minimizing strategy meant to contain pharmaceutical expenditure without compromising healthcare quality [6]. Several studies reported that substituting a branded drug for a generic drug can save money; such that, the use of generic medicines contributes in providing economical alternative to more expensive branded medicine and in reducing monetary expenditures [3, 7–9]. However, the safety and quality issues of generic products are of top concerns of general practitioners and health work professionals [10].

The increasing in healthcare costs is a global concern, in which drug expenditures represent a considerable percentage of these costs [11–13]. In Palestine, the total annual national health expenditure (THE) in 2008 was estimated to be US$ 893.8 million, which consisted of 15.6% of the Gross domestic product (GDP). The THE per capita was found to be US$ 165.5. Besides, the annual growth rate of total pharmaceutical market value was approximately 7%, while that of generic pharmaceutical market alone is 70% [14]. Therefore, highlighting the generic substitution policy as a cost containment strategy will lead to saving in healthcare budget in Palestine [15]. Since efficacy, safety and quality doubtful issues pertaining generic medicines, generic substitution and prescribing have been arguable among healthcare workers [16]. Not only developed countries, but also some developing countries were aware about the importance of adopting generic substitution strategies. According to the Palestinian pharmacy practice legislation, community pharmacists are not permitted to change or substitute prescribed medicines without the consent of the prescribing physician.

In the current study we would like to shed light on the rational of generic medicine use and to explore the criteria that Palestinian pharmacists based on for generic substitution. Besides, the question about promotion and acceptance of generic practices still needs to be addressed. However, no generic medicine or brand substitution prescribing policies implemented in Palestine. Several studies were conducted in different countries worldwide exploring the knowledge of pharmacist regarding generic medicines [1–3, 7, 11, 15, 17–24]. There are limited data about utilization of generics and generic substitution in Palestine, hence, it is important to explore the practicing pharmacists’ opinion and practice with regard to generic substitution issue. This study will help policy makers to implement suitable regulations regarding drug substitution policy to maximize the benefits on the patients and the Palestinian pharmaceutical practice. To the best of our knowledge there is no research surveyed community pharmacists in Palestine assessing their knowledge, perception, attitudes and practices towards generic medicine and substitution. As a result, pharmacists’ knowledge about generic substitution and dispensing plays an imperative role in enhancement generic medicine utilization. Therefore, the aim of the current study was to explore community pharmacists’ knowledge and to evaluate their attitudes and practices on generic medicines and substitution.

## Methods

### Study design

The current study surveyed a set of licensed community pharmacists who are members of Palestinian Pharmacists Syndicate using self-administered questionnaire. The study was carried out in a cross-sectional design.

### Participants, setting and sampling procedure

The study recruited practicing community pharmacists in the West Bank - Palestine. In Palestine, the majority of pharmacists work in private retail pharmacies called community pharmacies. Other forms of professional pharmacy in Palestine include pharmacists in industry, in regulatory governmental sector, and those in hospitals and other clinical settings. According to the Palestinian Pharmaceutical Syndicate, the West Bank is divided into 8 governorate sub-committees. These governorates are: East-Jerusalem, Ramallah, Bethlehem, Hebron, Jenin, Nablus, Tulkarm, and Qalqilia). A convenience sampling technique was implemented in this study. Different pharmacies located in cities, villages, and Palestinian refugee camps in West Bank were visited where practicing pharmacists were cordially invited to participate in the study. Participant pharmacists had to fill out the questionnaire in the presence of researcher and were not allowed to refer to any information resources during answering the questions. The data was collected from September 2016 to January 2017. Pharmacists affiliated with pharmacy hospitals or pharmacies affiliate with private clinical centers were excluded. Therefore, all participants were pharmacists in private community pharmacies.

### Sample size

The total number of licensed community pharmacies in West Bank (1027 pharmacies) was used as a guide to calculate the sample size (only one pharmacist from each pharmacy was interviewed). The estimated sample size was obtained using an automated online calculator (Raosoft sample size calculator: (http://www.raosoft.com/samplesize.html). It was used with pre-determined margin of error of 5%, and confidence level of 95%. The minimal approximated sample size was 280 pharmacists. In order to minimize erroneous results and to increase the study reliability, the target sample size was set to be about 300 pharmacists.

### Data collection form

A Self-administered questionnaire in the native Arabic language was conducted after the participants had agreed to participate (verbal consent form). A permission had been granted to use the data collection form as a tool which was developed and used by Awaisu and his co-authors [11]. The original questionnaire was in English. The questionnaire was translated by two colleagues who are American pharmacy graduates. The translated version was also re-checked by two different colleagues who are English Professors. The Arabic version included all statements in Arabic and in English. The questionnaire was divided into four sections (see Additional file 1). In the first section, questions on socio-demographic and professional characteristics were requested. The second part contains 10-item knowledge test statements on definition, and characteristics of generic medicines intended to examine the pharmacist’s knowledge. The respondent was given three options: “correct response,” “incorrect response” and “I do not know” to eliminate guessing. In scoring the pharmacists’ knowledge, a score of one was given to correct answers and score of zero was used for incorrect/do not know answers. The total scores range from 0 to 10 in which higher scores mean greater. The third section, pharmacists’ attitudes toward generic medicine was studied using a 15- item statements to explore their attitude toward generic medicines; regarding generic substitution policy for brand name medicines, and pharmacist s’ level of confidence. Participants had to respond on a 5- point Likert scale where, 1 indicated strong agreement and 5 indicated strong disagreement. No scoring was given to answers and all analysis was based on giving frequency for each response. In the fourth section, the influencing factors related to selection and dispensing of generic medicines was examined. With regard to validity of the tool, the face and content validity of the final questionnaire was discussed and judged by a panel of three specialist researchers/professors in pharmacy -identified as experts in generic medicine research and survey instrument development-for assessing the organization, clinical terminology, meaning of terms, completeness, appropriateness and logical sequence of the statements, and the accuracy [11]. The questionnaire was piloted among a sample of 25 pharmacists at the year of the study to test the reliability and validity of the questionnaire and to detect any defects in the methodology. The data of the pilot study were not included in the statistical analysis.

### Statistical analysis

Data were analyzed using Statistical Package for Social Sciences version 16 (SPSS 16). Frequencies and percentages of responses were generated for each answer in the questionnaire. Mann-Whitney-U test was used to detect difference in means of knowledge score between male and female participants, analyses of associations between categorical responses to compare means of knowledge score were tested using Kruskal-Wallis Test. *P*-values of less than 0.05 were considered statistically significant.

### Ethical approval

All aspects of the study protocol were authorized by the Institutional Review Board (IRB) of An-Najah National University (IRB- 11- July- 2016). Verbal consent was also obtained from the participant pharmacists prior to the commencement of the study.

## Results

### Demographic characteristics of participant pharmacists

A total of 339 community pharmacists were asked to participate in the study, however, 302 agreed to fill out the questionnaire with a response rate of 89.1%. Of the 302 pharmacists, 158 (52.3%) were males. The highest percent of participant pharmacists 128 (42.4%) were between the ages of (20–29) year-old, whereas few were above 59 years of age 14 (4.6%). The majority of pharmacists 260 (86.1%) had a B.Sc. in pharmacy whereas the rest had Pharm D or higher pharmacy degree. Most of the participants graduated from Palestinian universities 179 (59.3%). With regard to the experience, the majority 118 (39.1%) had 5 years or less of experience. Similarly, the years of practice in Palestine, the highest percent 124 (41.1%) were practicing 5 years. Regarding the classification of pharmacy’s location weather it is in a city, village or Palestinians refugee-camp, the results showed that most of the pharmacies 193 (63.9%) were concentrated in cities. With regard to job title of the participants they were divided equally to owner pharmacists and assistant pharmacists (employee). More details regarding the demographic and professional characteristics of the community pharmacists are presented in Table 1.

**Table 1:** Demographic characteristics of participant community pharmacists included in the study

Variable	*N* (%)
Gender	
Male	158 (52.3)
Female	144 (47.7)
Age	
20–29	128 (42.4)
30–39	81 (26.8)
40–49	59 (19.6)
50–59	20 (6.6)
>59	14 (4.6)
Education level	
Bachelor degree	260 (86.1)
Pharm D	28 (9.3)
Master degree	11 (3.6)
PhD	3 (1.0)
University of graduation	
Local	179 (59.3)
Regional	84 (27.8)
International	39 (12.9)
Years of experience	
5 or less	118 (39.1)
6–10	55 (18.2)
11–15	44 (14.6)
16–20	42 (13.9)
>20	37 (12.3)
Years of practice in Palestine	
5 or less	124 (41.1)
6–10	55 (18.2)
11–15	44 (14.6)
16–20	42 (13.9)
>20	37 (12.3)
Location of the pharmacy	
City	193 (63.9)
Village	99 (32.8)
Palestinians refugee-camps	10 (3.3)
Job title	
Owner	151 (50.0%)
Employee	151 (50.0%)

### Pharmacists’ knowledge about generic medicine

Ten knowledge items were tested, the median score of knowledge on generic medicines of the participants was found to be 6.00 (inter quartile range: 5.00–7.00) (mean ± SD: 5.91 ± 1.27). The association of demographic and professional characteristics on knowledge score was analyzed using Mann-Whitney-U and Kruskal-Wallis tests. Table 2 clearly shows that there were no statistically significant differences in knowledge score between participant pharmacists according to the eight socio-demographic characteristics examined (*p*-values of more than 0.05). Detailed findings regarding knowledge scoring among different demographic features of the respondents are given in Table 2. The highest knowledge score value was 8 out 10 that obtained by 26 (8.6%) of respondent pharmacists, while 2 out 10 scored the lowest scale.

**Table 2:** Association between socio-demographic characteristics of participant pharmacists’ and knowledge score regarding generic medicine

Characteristic	Median (Q1-Q3)	Mean ± SD	*P*-value
Gender			
Male	6 (5–7)	5.92 ± 1.30	0.971
Femal**e**	6 (5–7)	5.91 ± 1.24	
Age			
20–29	6 (5–7)	5.86 ± 1.22	0.734
30–39	6 (5–7)	5.93 ± 1.31	
40–49	6 (5–7)	6.03 ± 1.19	
50–59	6 (5–6.75)	5.75 ± 1.29	
>59	7 (5_7.25)	6.07 ± 1.77	
Academic degree			
B.Sc.	6 (5–7)	5.92 ± 1.28	0.975
Pharm D	6 (5–7)	5.93 ± 1.12	
M.Sc.	6 (5–7)	6.00 ± 1.18	
Ph.D.	6 (3)	5.00 ± 1.73	
University of graduation			
Locally	6 (5–7)	5.94 ± 1.16	0.235
Regional	6 (5–7)	5.74 ± 1.46	
Internationally	6 (5–7)	6.18 ± 1.30	
Years of experience			
5 or less	6 (5–7)	5.84 ± 1.21	0.737
6–10	6 (5–7)	5.87 ± 1.26	
11–15	6 (5–7)	5.96 ± 1.38	
16–20	6 (5–7)	6.07 ± 1.24	
More than 20	6 (5–7)	5.98 ± 1.37	
Years of practice			
5 or less	6 (5–7)	5.85 ± 1.21	0.715
6–10	6 (5–7)	5.85 ± 1.30	
11–15	6 (5–7)	6.14 ± 1.29	
16–20	6 (5–7)	5.90 ± 1.28	
More than 20	6 (5–7)	5.95 ± 1.41	
Location of pharmacy			
City	6 (5–7)	5.86 ± 1.36	0.664
Village	6 (5–7)	6.04 ± 1.09	
Palestinians Refugees Camps	5.5 (5–7)	5.70 ± 1.06	
Job title			
Pharmacy owner	6 (5–7)	5.95 ± 1.23	0.691
Pharmacist assistant	6 (5–7)	5.87 ± 1.30	

High percent of the respondents pharmacists 286 (94.7%) answered correctly item 4 in Table 3 which revolved about presumptuous similarity in efficacy, quality and safety aspects when bioequivalency is conquered*.* Similarly, 280 (92.7%) of participant pharmacists answered correct response to item 2 in Table 3 which focused on the demands of bioequivalence studies for generic registration approval. Of the 302 participant 251 (83.1%) pharmacists responded correctly to item 5 in Table 3 which questioned the consequences of bioequivalent studies and their impacts. However, item 6 which probed the FDA acceptance criteria in statistical comparative analysis of the pharmacokinetic parameter to be assessed in bioequivalence study [25], only 38 (12.6%) community pharmacists provided correct answer. Likely, item 8 that stated “*when two pharmaceutical products are bioequivalent, it means that the C*_*max*_ *and AUC ratios estimated for each formulation can vary by -20 to +25%”* [25]*,* few of the participants 68 (22.5%) answered correctly. Detailed responses on knowledge items are provided in Table 3.

**Table 3:** Knowledge of community pharmacists in Palestine on generic medicine (*n* = 302)

Knowledge item	Correct response *N* (%)	Incorrect response *N* (%)	I don’t know *N* (%)
1. The term generic medicine is a drug product marketed under the drugs non-proprietary approved name or a product marketed under a different brand name (proprietary) name. (T)	192 (63.6)	93 (30.8)	17 (5.6)
2. Generic products must be bioequivalent to the innovator brand before they can be approved to be marketed in many developed and some developing countries. (T)	280 (92.7)	9 (3.0)	13 (4.3)
3. Product quality data are NOT required before a generic product can be registered in such countries that require bioequivalent data. (F)	21 (7.0)	239 (79.1)	42 (13.9)
4. Provided that a generic product conforms to bioequivalence and product quality requirements, it is assumed that its efficacy, quality and safety are similar to the original branded product. (T)	286 (94.7)	13 (4.3)	3 (1.0)
5. Two pharmaceutical products are bioequivalent if they are pharmaceutically equivalent and their bioavailabilities are similar to such a degree that their effects, with respect to both efficacy and safety, can be expected to be essentially the same. (T)	251 (83.1)	23 (7.6)	28 (9.3)
6. For generic drug to be bioequivalent to its innovator brand or other generics, the 90% confidence intervals for the ratio of each pharmacokinetics parameters (i.e. C_max_ and AUC), must lie within the range of 90–110%. (F)	156 (51.7)	38 (12.6)	108 (35.8)
7. A generic medicine is usually manufactured without a license from the innovator company, but marketed after expiry of patent or other exclusivity rights. (T)	179 (59.3)	87(28.8)	36 (11.9)
8. When two pharmaceutical products are bioequivalent, it means that the Cmax and AUC ratios estimated for each formulation can vary by −20 to +25%. (T)	68 (22.5)	94(31.1)	140 (46.4)
9. Where there is a generic substitution policy, the community pharmacists is allowed to dispense a different brand of the drug, but may or may not refer back to the prescriber depending on the jurisdiction/law. (F)	263 (87.1)	23 (7.6)	16 (5.3)
10. If a generic medicine is bioequivalent to a branded medicine, it means that it is also therapeutically equivalent. (T)	231 (76.5)	56(18.5)	15 (5.0)

### Pharmacists’ attitudes toward generic medicines

Table 4 obviously showed that the majority of participant pharmacists disagreed 3 attitudinal items out of 15. These are: item 4, 5 and 15 which illustrated the switching from brand to generic, reporting of therapeutic failure as a major problem in most generics and stopping of dispensing generics issues respectively. Detailed results are listed below and are provided in Table 4. Out of 302 respondents 188 (62.3%) agreed to attitudinal item 1 in Table 4 which explored the pharmacists’ supportiveness of generic substitution policy. In addition, 168 of the participants (55.6%) agreed item 2 that argued the lowering spending consequences on developmental research on discovering new medication that may result from using generic versions of drugs, while 76 (25.1%) of the pharmacists disagreed this point. Approximately two third of the participants (68.2%) agreed item 3 that linked reduction of governmental health-care expenditure to wider use of generics. A little more than half of participant pharmacists 157 (52.0%) disagreed item 4 that covered the issue of changing in therapeutic outcomes when a brand is switched to a generic. The majority of the pharmacists 227 (75.2%) disagreed the statement “*therapeutic failure is a serious problem with most generic products”*. When the pharmacists were asked when considering generic medicines as therapeutically equivalent products, the majority 219 (72.5%) agreed this item (6) in Table 4. About two third (67.6%) of pharmacists who asked about item 7 which elucidated the differences in prices between branded and generic versions as a reason for substitution. When participants were asked in item 8 about patients’ right of taking adequate clarifications why generic medicines were chosen, 246 (81.4%) pharmacists were agreed the point. Most of the pharmacists 264 (87.4%) said that community pharmacists in Palestine should be given generic substitution right. A majority of participant pharmacists (90.4%) believed that generic dispensing is significantly influenced by the intensity of promotional activities by medical representatives (item 10). Most of included pharmacists in the study 288 (95.4%) agreed item 11 in Table 4 which talked about the necessity of applying policies by health authorities like bioequivalence studies prior to marketing approval of generic product, while 7 (2.3%) disagreed the point. Item 12 which stated “*community pharmacists should be allowed to perform generic substitution without consulting the prescribing physician*” 69.5% agreed this attitudinal item. Almost half of participants 148 (49.0%) disagreed item 13 in Table 4 which concentrated on prescribers’ consultation by pharmacists prior of engaging in generic substitution. A large number of participant pharmacists 195 (64.5%) suggested that community pharmacists need to refer to prescriber in special cases of generic substitution such as substitution of drugs with narrow therapeutic indices (item 14). One hundred and seventy two (57.0%) of pharmacists disagreed the statement “*in general, I would not dispense generic medicine to my patients*” (item 15) in Table 4.

**Table 4:** Participant pharmacists’ attitudes on generic medicine in Palestine (*n* = 302)

Attitudinal item	Strongly agree *N* (%)	Agree *N* (%)	Neutral *N* (%)	Disagree *N* (%)	Strongly disagree *N* (%)
1. I support generic substitution for brand name drugs in all cases where a generic is available.	66 (21.9)	122 (40.4)	17 (5.6)	85(28.1)	12 (4.0)
2. Wider use of generic medicines will mean that less money will be spent for research and development of new pharmaceuticals.	38 (12.6)	130(43.0)	58 (19.2)	69(22.8)	7 (2.3)
3. Wider use of generic medicines will result in decrease in health care expenditure by the government of Palestine.	51 (16.9)	155 (51.3)	46 (15.2)	43(14.2)	7 (2.3)
4. Switching a patient from branded medicine to a generic medicine may change the outcome of the drug therapy.	16 (5.3)	75 (24.8)	54 (17.9)	132(43.7)	25 (8.3)
5. Therapeutic failure is a serious problem with most generic products.	13 (4.3)	27 (8.9)	35 (11.6)	176(58.3)	51 (16.9)
6. All products approved as generic drugs by the health authorities in the state of Palestine can be considered therapeutically equivalent to their branded counterparts.	48 (15.9)	171 (56.6)	35 (11.6)	42(13.9)	6 (2.0)
7. The price difference between generic and branded drugs is often so great that I feel I must dispense prescriptions with generic substitution, especially for people who do not have prescription drug benefits in Palestine.	57 (18.9)	147 (48.7)	18 (6.0)	68(22.5)	12 (4.0)
8. Patients should be given enough explanations about the reasons for choosing generic medicines for them.	75 (24.8)	171 (56.6)	17 (5.6)	31(10.3)	8 (2.6)
9. Community pharmacists in Palestine should be given generic substitution right.	117 (38.7)	147 (48.7)	16 (5.3)	17 (5.6)	5 (1.7)
10. The intensity of promotional activities by medical representatives plays an important role in dispensing generics.	99 (32.8)	174 (57.6)	9 (3.0)	16 (5.3)	4 (1.3)
11. Health authorities in Palestine should implement policies such that bioequivalence data are mandatory before a generic product is marketed.	135 (44.7)	153 (50.7)	7 (2.3)	6 (2.0)	1 (0.3)
12. Community pharmacists should be allowed to perform generic substitution without consulting the prescribing physician.	91 (30.1)	119 (39.4)	30 (9.9)	49(16.2)	13(4.3)
13. Community pharmacists must consult the prescribing physician when performing generic substitution.	23 (7.6)	97 (32.1)	34 (11.3)	121(40.1)	27 (8.9)
14. Community pharmacists should only be required to consult the prescribing physician when substituting certain categories of drugs, such as those with narrow therapeutic index.	65 (21.5)	130 (43.0)	39 (12.9)	62(20.5)	6 (2.0)
15. In general, I would not dispense generic medicine to my patients.	19 (6.3)	67 (22.2)	44 (14.6)	122(40.4)	50 (16.6)

### Influencing factors linked to selection and dispensing of generic medicines

Table 5 illustrates possible influencing factors related to selection and dispensing of generic medicines among the community pharmacist. The majority of pharmacists reported that the two main factors affecting their selections and dispensing generic medicines are personal faith in the product and cost effectiveness of generic medicines 260 (86.1%) and 254 (84.1%) respectively.

**Table 5:** Possible influencing factors related to selection and dispensing of generic medicines among the community pharmacist

Factor	Important influencing factor *N* (%)	Neutral *N* (%)	Unimportant influencing factor *N* (%)
Lack of belief in generic medicines	217 (71.9)	50 (16.6)	35 (11.6)
Availability of policies,law and regulations	188 (62.3)	76 (25.2)	38 (12.6)
Legal implication	167 (55.3)	98 (32.5)	37 (12.3)
Cheaper cost to the customer	241 (79.8)	34 (11.3)	27 (8.9)
Having no other choice	217 (71.9)	59 (19.5)	26 (8.6)
Consumer preference /demand	217 (71.9)	51 (16.9)	34 (11.3)
Availability of stock	219 (72.5)	43 (14.2)	40 (13.2)
Customer’s appearance or nationality	84 (27.8)	76 (25.2)	142 (47.0)
Cost effectiveness of generic medicines	254 (84.1)	26 (8.6)	22 (7.3)
Data or information about proven bioequivalence to original brand	216 (71.5)	55 (18.2)	31 (10.3)
Personal faith in the product	260 (86.1)	23 (7.6)	19 (6.3)
Substitution agreement with prescriber	228 (75.5)	51 (16.9)	23 (7.6)

## Discussion

### Pharmacists’ knowledge about generic medicines

In general, participant pharmacists had relatively low knowledge score on generic medicines. However, our data showed that the majority of participant pharmacists had enough information about the definition of generics. Similarly, almost two thirds of pharmacists in Poland correctly answered the term assessing the definition of generic medicines [23]. These results are harmonic with former studies that have been recorded from other improving nations, such as Pakistan and Malaysia [6, 15].

Although the mean knowledge score of the respondents about generic medicines were lower than Qatar’s survey findings, our data were similar to those reported by Awaisu et al. in the following aspects; (i) poor knowledge of pharmacists included in both countries in the area of bioequivalence notions, the pharmacokinetic parameters (i.e., AUC, C_max_) and the technical determination of bioequivalent products. (ii) no significant association between knowledge score on generic medicines and the socio-demographic and professional characteristics. However, the obtained results assessing the therapeutic equivalency were higher compared to results reported by Awaisu and his colleagues [11]. These feedbacks suggest a demand for interventions to develop pharmacists’ awareness on these important matters underlining the safety, quality and efficacy of generic medicines.

Similarly, In Saudi Arabia, Wajid et al. [18] revealed that community health practitioners own doubts on curative efficacy and integrity profiles of generic medicine products. This outcome could be linked to their poor awareness about the notion of bioequivalence, the FDA acceptance criteria and the statistical comparative analysis of the pharmacokinetic parameter to be assessed in bioequivalence study.

Drozdowska and Hermanows [23] clarified that characteristics like: sex, age, pharmacy case (series pharmacy vs. separate pharmacy), and site of pharmacy did not distinguish the pharmacists answers to a statistically considerable level in referring to repetition that made patients aware that they have a choice of generic substitution (*p* > 0.05), and this outcome is similar to our study in Palestine. However, a survey conducted in New Zealand reported a significant variation in pharmacists’ awareness of generic medicines based on the length of time in expertise; the participants with less than 5 years in expertise had a better perception of generic drugs than those with larger than or similar to 5 years in expertise [24].

### Pharmacists’ practice and attitude toward generic medicines

The current study showed that among factors affecting the pharmacists’ generic drug exchange experiences according to the participants contain the cheaper price to the customer, faith in the product, having no other options and cost-efficiency of the generic drugs. These findings were similar to previous studies that reported these agents as substantial influencing agents [11, 18, 23, 24]. In addition, the majority of the respondents agreed that the patient should have right of having adequate information on the use of generic medicines. In Poland, less than half of the participants introduced consumers about generic product [23].

The overall pharmacists’ attitudes on generic medicines in our survey in Palestine was similar to former study in Saudi Arabia accomplished by Wajid et al. [18], where participants were positively directing to generic exchange as they see it as definition of their function as drug treatment experts [18].

Our data announced that the plurality of the participants united in opinion on that the community pharmacist should have the right to perform generic substitution without consulting the prescribing physician. This was similar to results reported in Qatar [11]. But, O’Leary et al. [20] reported that the matters mostly concentrated on medications with limited therapeutic indices i.e. antiepileptic medications, digoxin, warfarin and thyroxine in addition to sustained-released and multi-constituent forms. In some civilized counties such as Canada and New Zealand, there are a finite number of drugs that are believed non-interchangeable drugs, and these drugs are exempt from generic exchange.

## Conclusions

In conclusion, this study showed that a large proportion of participant community pharmacists in in Palestine had basic knowledge with regards to generic medicines. However, their knowledge score pertaining to the technical and regulatory aspects of bioequivalence and pharmacokinetic parameters, in particular, were relatively low. The results also showed that most of the participants were supportive to generic drug substitution policy. Participants showed a positive attitude towards generic medicines and generic substitution in Palestinian community pharmacies. Better knowledge and perception of generic medicines is very important in establishing and adopting a national generic medicine policy and guidelines. The increasing number of generic medicines and popularity necessitate regulatory bodies to to increase awareness of pharmacists about generics. Besides, there is a demand to encourage practicing pharmacists to attend more structured training schedule about generic medicines. The role of pharmacists in providing patients with information about the generic products emphasizes their crucial participation in communicating with patients and be aware of generic products safety, efficacy and quality issues.

### Limitations

Since the method recruit a self-administered questionnaire, response bias is likely. The use of self-evaluation and limited questions for evaluation of knowledge are limitation of this research. Normally, the best way for practice evaluation is achieved through observation. In addition, the generalization of the results is limited as the sample of pharmacists was taken from West Bank alone which may not be representative of all Palestinian pharmacists. Besides, the study did not include biosilmalr products since they are not available in the Palestinian market.

## Additional file

Additional file 1: The data collection form in English. (DOCX 44 kb)

## Abbreviations

AUC: area under the concentration-time curve
C_max_: maximum plasma drug concentration
FDA: Food and Drug Administration
GDP: gross domestic product
INN: international nonproprietary names
IRB: institutional review board
THE: the total annual national health expenditure
USA: United State America
