# Supplementary material for: Knowledge, attitudes and practices of community pharmacists on generic medicines in Palestine: a cross-sectional study
Source: BMC Health Serv Res. 2017 Dec 28;17:847. doi: 10.1186/s12913-017-2813-z (PMC5745619; doi:10.1186/s12913-017-2813-z)
Supplement: Additional file 1: — The data collection form in English. (DOCX 44 kb) [file 12913_2017_2813_MOESM1_ESM.docx]

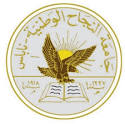


**Research Questionnaire**

**An - Najah National University**

**Department of Pharmacy**

**Dear pharmacists who are working in community pharmacies in Palestine you are cordially invited to participate in the research and to fill out the current questionnaire aimed to assess the knowledge of pharmacists in Palestine, their attitudes and practices towards generic medicines**

**You have full freedom and will to participate in this research and you have the right to take the time to think about participation or not and ask the researcher what it deems appropriate and talk to any person or entity about this research.**

**You can also inquire about any part of the search now or later. If there are words or parts that are not understood, you can ask the researcher and you will find the correct time and answer.**

**We confirm that all information collected from you is for scientific research purposes only and will be kept strictly confidential and will not be used for other purposes.**

**Thank you for your efforts and time to answer the questions**

**Section I: Demographic characteristics of community pharmacist in Palestine:**

**Female Gender: Male**

**not Palestinian Palestinian : Nationality**

**Age*:* ……………**

**Educational level:** **Bachelor degree Pharm D Master degree PhD**

**University of graduation: ……………………. country of graduation ………………..**

**Years of experience: …………………………………**

**Years of practice in Palestine:……………..**

**Location of the pharmacy: City village Palestinians Refugees Camps**

Pharmacy assistant

**The governorates where the pharmacy is located: ……………………………….**

**Pharmacy assistant Job title: Pharmacy owner**

Pharmacy assistant

**Section II: Knowledge of community pharmacists in Palestine on generic medicines. Please answer the following sentences ticking (√ )in the appropriate box**

| **knowledge item** | **Correct** | **Incorrect** | **I am not sure** |
| --- | --- | --- | --- |
| 1.The term generic medicine is a drug product marketed under the drug’s non-proprietary approved name or a product marketed under a different brand name (proprietary) name |  |  |  |
| 2. Generic products must be bioequivalent to the innovator brand before they can be approved to be marketed in many developed and some developing countries. |  |  |  |
| 3. Product quality data are NOT required before a generic product can be registered in such countries that require bioequivalent data. |  |  |  |
| 4. Provided that a generic product conforms to bioequivalence and product quality requirements, it is assumed that its efficacy, quality and safety are similar to the original branded product. |  |  |  |
| 5.Two pharmaceutical products are bioequivalent if they are pharmaceutically equivalent and their bioavailabilities are similar to such a degree that their effects, with respect to both efficacy and safety , can be expected to be essentially the same |  |  |  |
| 6. For generic drug to be bioequivalent to its innovator brand or other generics, the 90% confidence intervals for the ratio of each pharmacokinetics parameters (i.e. Cmax and AUC), must lie within the range of 90-110%. |  |  |  |
| 7. A generic medicine is usually manufactured without a license from the innovator company, but marketed after expiry of patent or other exclusivity rights. |  |  |  |
| 8. When two pharmaceutical products are bioequivalent , it means that the Cmax and AUC ratios estimated for each formulation can vary by -20 to +25% |  |  |  |
| 9. Where there is a generic substitution policy, the community pharmacists is allowed to dispense a different brand of the drug, but may or may not refer back to the prescriber depending on the jurisdiction/law. |  |  |  |
| 10. If a generic medicine is bioequivalent to a branded medicine, it means that it is also therapeutically equivalent. |  |  |  |

**Section III: The following table represents the pharmacists’ attitudes in the community about the investment of generic medicines in Palestine. Please rate the following sentences ticking (√ ) in the appropriate box:**

| **Strongly disagree** | **disagree** | **Neutral** | **Agree** | **Strongly agree** | **Attitudinal item** |
| --- | --- | --- | --- | --- | --- |
|  |  |  |  |  | 1. I support generic substitution for brand name drugs in all cases where a generic is available. |
|  |  |  |  |  | 2. Wider use of generic medicines will mean that less money will be spent for research and development of new pharmaceuticals. |
|  |  |  |  |  | 3. Wider use of generic medicines will result in decrease in health care expenditure by the government of Palestine. |
|  |  |  |  |  | 4. Switching a patient from branded medicine to a generic medicine may change the outcome of the drug therapy |
|  |  |  |  |  | 5. Therapeutic failure is a serious problem with most generic products. |
|  |  |  |  |  | 6. All products approved as generic drugs by the health authorities in the state of Palestine can be considered therapeutically equivalent to their branded counterparts. |
|  |  |  |  |  | 7. The price difference between generic and branded drugs is often so great that I feel I must dispense prescriptions with generic substitution, especially for people who do not have prescription drug benefits in Palestine. |
|  |  |  |  |  | 8. Patients should be given enough explanations about the reasons for choosing generic medicines for them. |
|  |  |  |  |  | 9. Community pharmacists in Palestine should be given generic substitution right |
|  |  |  |  |  | 10. The intensity of promotional activities by medical representatives plays an important role in dispensing generics. |
|  |  |  |  |  | 11. Health authorities in Palestine should implement policies such that bioequivalence data are mandatory before a generic product is marketed. |
|  |  |  |  |  | 12. Community pharmacists should be allowed to perform generic substitution without consulting the prescribing physician |
|  |  |  |  |  | 13. Community pharmacists must consult the prescribing physician when performing generic substitution |
|  |  |  |  |  | 14. Community pharmacists should only be required to consult the prescribing physician when substituting certain categories of drugs, such as those with narrow therapeutic index. |
|  |  |  |  |  | 15. In general, I would not dispense generic medicine to my patients. |

**Section IV: Possible influencing factors related to selection and dispensing of generic medicines among the community pharmacists please answer the following sentences ticking (√ ) in the appropriate box :**

| **Unimportant influencing factor** | **Neutral** | **Important influencing factor** | **Factor** |
| --- | --- | --- | --- |
|  |  |  | 1. Lack of belief in generic medicines |
|  |  |  | 2. Availability of policies ,law and regulations |
|  |  |  | 3. Legal implication |
|  |  |  | 4. Cheaper cost to the customer |
|  |  |  | 5. Having no other choice |
|  |  |  | 6. Consumer preference /demand |
|  |  |  | 7. Availability of stock |
|  |  |  | 8. Customer's appearance or nationality |
|  |  |  | 9. Cost effectiveness of generic medicines |
|  |  |  | 10. Data or information about proven bioequivalence to original brand |
|  |  |  | 11. Personal faith in the product |
|  |  |  | 12. Substitution agreement with prescriber |

**Thank you for your time and cooperation**
